# Synovial sarcoma with intra-abdominal metastasis causing hemoperitoneum: a case-report

**DOI:** 10.1186/s12957-023-02885-2

**Published:** 2023-01-30

**Authors:** Joshua J. Thompson, Rachel A. Koch, Vicki L. Keedy, Deepa R. Magge

**Affiliations:** 1 Department of Surgery, Vanderbilt University Medical Center, Preston Research Building, 2220 Pierce Avenue, Nashville, Tennessee 37232-6860 USA; 2 Vanderbilt-Ingram Cancer Center, Vanderbilt University Medical Center, Nashville, Tennessee USA; 3 Department of Surgical Oncology, Vanderbilt University Medical Center, Nashville, Tennessee USA

**Keywords:** Synovial sarcoma, Screening, Metastasis

## Abstract

Synovial sarcoma is a rare soft tissue sarcoma which frequently involves the upper or lower extremities. Soft tissue sarcomas including synovial sarcoma have a propensity to metastasize to the lungs, and there are very few reports of metastatic lesions in other locations.

Here, we report a case of a 49-year-old patient who underwent neoadjuvant chemoradiation for an upper extremity synovial sarcoma and presented approximately 4 years later with abdominal pain and hemoperitoneum and was ultimately found to have metastatic synovial sarcoma involving the greater curvature of the stomach and surrounding peri-gastric soft tissue. We describe the multidisciplinary management of this complex patient presentation and propose that expanded surveillance imaging beyond that of the local tumor resection bed and the chest may be beneficial especially in tumors with high-risk features.

## Background

Synovial sarcoma is a rare and malignant type of soft tissue sarcoma often affecting the upper or lower extremities with only approximately 800 to 1000 cases per year in the USA [1]. Once thought to be more common in young adults, it is commonly seen in patients aged 20–49 years of age with some studies noting a peak incidence in patients 45–49 years old [2]. The 5-year survival rate in adults ranges from 50 to 60% and the 5-year metastatic disease-free survival is anywhere from 40 to 60% [3]. Pediatric patients with synovial sarcoma tend to have significantly better outcomes with some studies reporting a 90% 5-year survival rate [4, 5].

These tumors are driven by SS18:SSX fusion oncogenes generated from the chromosomal translocation of the SS18 gene which codes for a subunit of the BAF chromatin remodeling complex (SWI/SNF complex) with the transcriptional repressors SSX1, SSX2, or SSX4 (synovial sarcoma, X breakpoint genes) which ultimately disrupts global chromatin remodeling and has noted downstream effects on key oncogenic signaling pathways involving E-cadherin, Bcl-2, Mcl1, and Wnt [6, 7].

Staging of synovial sarcomas (as with the majority of other soft tissue sarcomas located on the trunk or extremities) is based on standard TNM definitions with the addition of a histologic grading scale as follows: T1 tumors are < 5 cm in greatest dimension, T2 tumors are 5–10 cm, T3 tumors are 10–15 cm, and T4 tumors are > 15 cm in greatest dimension. N0 denotes no regional lymph node metastasis or unknown lymph node status while N1 tumors demonstrate regional lymph node metastasis. M0 denotes no distant metastasis while M1 tumors display distant metastasis. Histologic grading is based on the Fédération nationale des centres de lutte contre le cancer (FNCLCC) grading system and factors in the following: tumor differentiation (synovial sarcomas frequently display significant dedifferentiation), mitotic count (0–9 mitoses per HPF, 10–19 mitosis per 10 HPF, >20 mitosis per HPF), and % tumor necrosis (no necrosis, < 50% tumor necrosis, > 50% tumor necrosis). Further histologic subtyping of synovial sarcoma includes monophasic spindle cell, biphasic (with spindle cells and gland-like epithelial structures), and poorly differentiated tumors (more round cell morphology) [8].

Treatment of synovial sarcoma involves a multi-disciplinary team approach with wide surgical excision as the mainstay of treatment when acceptable functional outcomes following resection are achievable. Per National Comprehensive Cancer Network (NCCN) guidelines, patients with Stage II disease can proceed directly to surgery or undergo preoperative radiotherapy while patients with Stage III disease should be considered for preoperative radiotherapy ± preoperative systemic therapy [8]. Per NCCN guidelines, follow-up includes history and physical examination every 3–6 months for 2–3 years then every 6 months for the next 2 years and then annually. Chest imaging and periodic imaging of the primary site based on estimated risk of locoregional recurrence is also recommended. Of note, for extremity, body wall, and head and neck soft tissue sarcomas, there are currently no guidelines or recommendations for routine surveillance of the abdomen or pelvis and reports of intra-abdominal metastasis are quite rare.

## Case presentation

The patient is a 53-year-old female with a past medical history of hypertension, hyperlipidemia, and gastroesophageal reflux disease who presented approximately 4 years following resection of a right forearm synovial sarcoma with a gastric mass concerning for metastatic synovial sarcoma.

Her oncologic history is notable for a 7.6 x 5.3 x 2.6 cm right dorsal forearm synovial sarcoma diagnosed in January 2017, at age 49, on core biopsy (FNCLCC Grade 2, 12 mitotic figures per HPF, no necrosis, pancytokeratin positive, negative for MUC4, SOX 10, Desmin, myogenin, and myoD1 with focal weak SMA immunoreactivity, SS18/SSX1 fusion positive). She underwent neoadjuvant chemoradiation with 3 cycles of doxorubicin (75 mg/m^2^) and ifosfamide (10,000 mg/m^2^) (complicated by mucositis, pancytopenia, and neutropenic fevers with cycle 3) and local radiation followed by wide resection 7 months after the initial diagnosis. Final pathology demonstrated monophasic synovial sarcoma with treatment effect, 75% viable residual tumor, and negative margins with the closest margin being 0.2 cm. Her postoperative course was unremarkable, and she was discharged on POD1. She subsequently underwent two additional cycles of adjuvant doxorubicin and ifosfamide postoperatively.

She underwent routine surveillance imaging including upper extremity MRI with and without contrast and a chest CT approximately every 6 months postoperatively. At 1-year postop, the patient was without evidence of recurrent disease. She continued with screening chest X-ray and forearm X-rays alternating every 6 months with upper extremity MRI and chest CTs. Prior to her hospital presentation, she was seen in 06/2020 (3 years post-resection) without evidence of recurrent disease in the arm or metastatic disease in the chest.

**Fig. 1:**
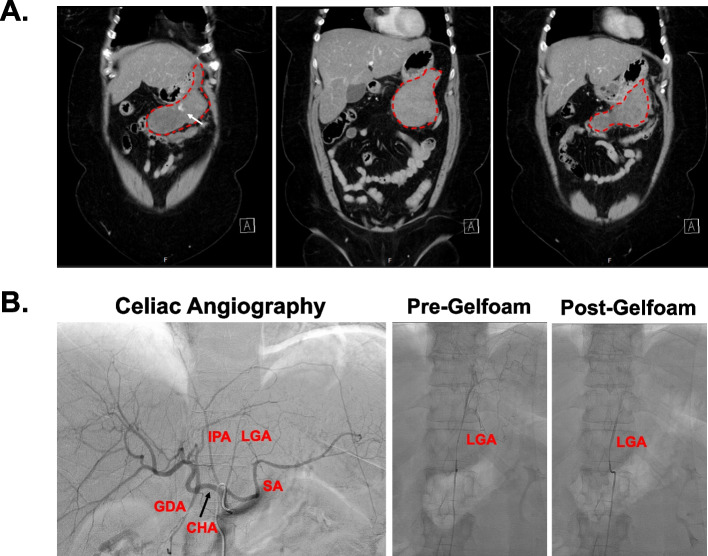
**A** 43 months after primary resection, patient presented with epigastric abdominal pain and tachycardia. Multiple coronal slices of CT demonstrate ~ 6.8 x 6.1 x 5.4 cm mass along greater curvature of the stomach (red outline) with associated active extravasation (white arrow). **B** Angiogram and gelfoam embolization of left gastric artery (LGA). Celiac angiography identifies diminutive splenic artery (SA), left gastric artery (LGA), inferior phrenic artery (IPA), gastroduodenal artery (GDA), and common hepatic artery (CHA)

**Fig. 2:**
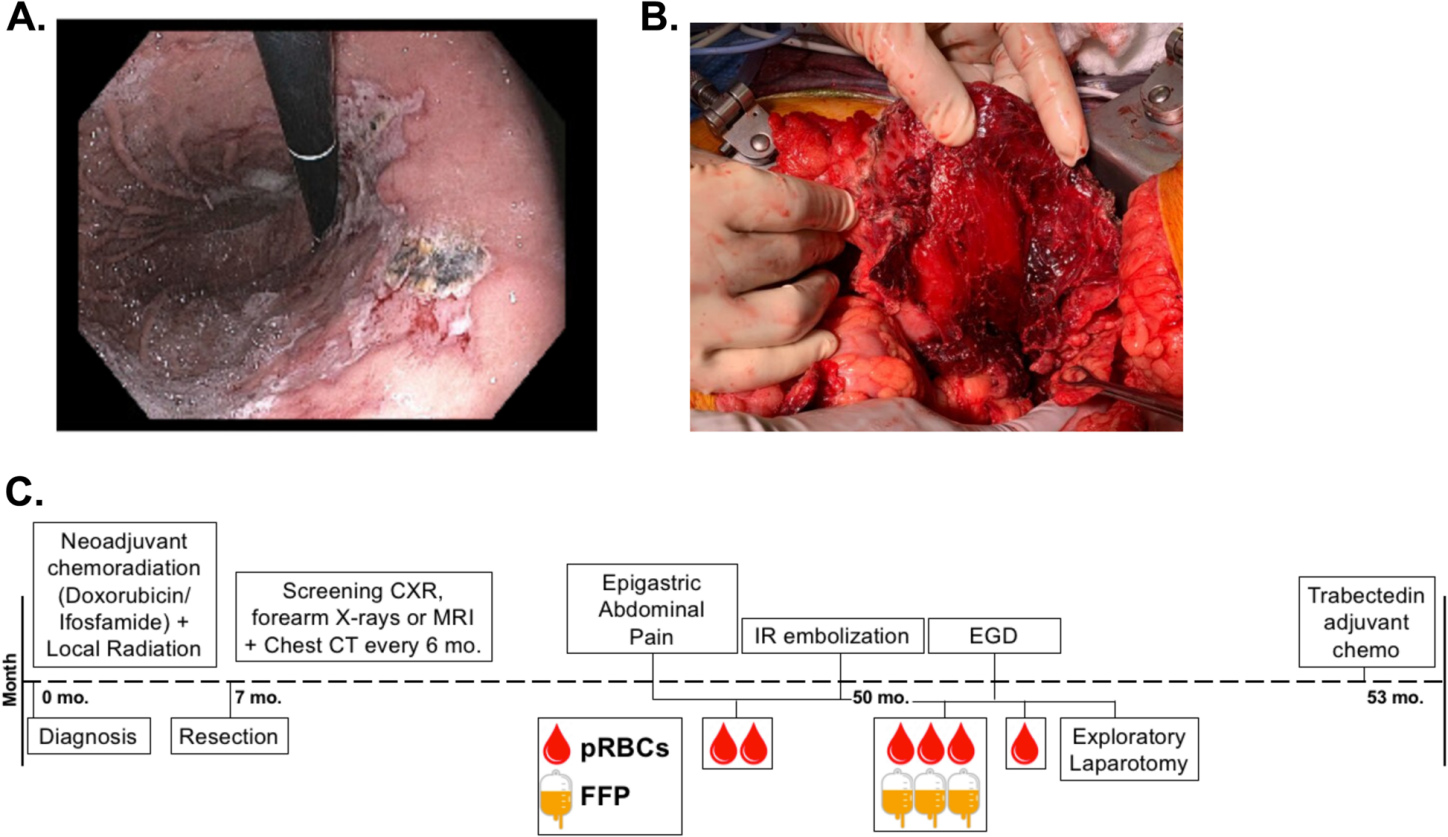
**A** EGD demonstrated superficial gastric ulceration in the gastric body and greater curvature of the stomach. Endosonographically this mass was hypoechoic and heterogeneous with poorly defined borders admixed with amorphous hematoma. FNA of this mass was obtained. **B** Intra-operative images demonstrate significant adherence of the greater curve of the stomach to the transverse colon. The lesser sac was obliterated and found to be full of hematoma with significant deserosalization of the posterior wall of the stomach secondary to perforated tumor with adherence of the pancreatic tail to the proximal and lateral gastric walls along with subserosal hematoma tracking proximally to the gastroesophageal junction. **C** Timeline of treatments and interventions. Transfusions of packed red blood cells (pRBCs) and fresh frozen plasma (FFP) indicated

She then presented from an outside hospital in February 2021 with epigastric abdominal pain and tachycardia and was found to have a 6.8 x 6.1 x 5.4 cm mass along the inferomedial greater curvature of the stomach with concern for active extravasation and associated hemoperitoneum (Fig. 1A). She denied any episodes of hematemesis, hematochezia, or melena. She received 2 units of pRBCs en-route and remained tachycardic with heart rates in the low 120s but normotensive with systolic blood pressures in the 120s. A repeat CT Angiogram of the abdomen and pelvis was obtained which demonstrated a pseudo-vein sign consistent with active contrast extravasation, and the patient was subsequently taken to the procedure suite by interventional radiology for sub-selective gelfoam embolization of the left and right gastric arteries (Fig. 1B). She was admitted to the medical ICU post procedurally for ongoing resuscitation and ultimately received an additional 3 units pRBCs and 3 units FFP for persistent tachycardia. Given the appearance of the lesion on imaging, there was concern that this mass may represent a gastrointestinal stromal tumor (GIST) versus a metastatic lesion from her original sarcoma. Gastroenterology was consulted for EGD and EUS/FNA. On EGD, the patient was noted to have superficial gastric ulceration in the gastric body and greater curvature of the stomach (Fig. 2A). On endoscopic ultrasound, patient noted to have an irregular, hypoechoic, and heterogeneous mass with poorly defined borders extending from the gastric wall into the perigastric peritoneal space. FNA of this lesion was performed which demonstrated a small amount of small round blue cells in abundant myxoid matrix (negative for synaptophysin, chromogranin, calponin, SMA, CD117, DOG1, CD34, S100, and cytokeratin staining using AE1/AE3 cocktail (CK1–8, 10, 14–16, and 19). Of note, this sample was compared to the patient’s synovial sarcoma and showed minimal significant cytologic similarity. Post-procedurally, the patient was persistently tachycardic to the 120s with a new oxygen requirement of 1–2 l nasal canula and a concomitant drop in her hemoglobin from 8.8 to 6.8 requiring transfusion of an additional 1-unit pRBCs. She underwent a CT angiogram pulmonary embolism protocol which was negative for pulmonary embolus but did demonstrate an interval enlargement of the peri-gastric hematoma with active extravasation into the greater curvature of the stomach.

Given the concern for ongoing bleeding, the patient was taken to the operating room for exploration. On diagnostic laparoscopy, significant hemoperitoneum and poor visualization prompted conversion to an exploratory laparotomy. There was significant adherence of the greater curve of the stomach to the transverse colon. The lesser sac was obliterated and found to be full of hematoma with significant deserosalization of the posterior wall of the stomach secondary to perforated tumor with adherence of the pancreatic tail to the proximal and lateral gastric walls along with subserosal hematoma tracking proximally to the gastroesophageal junction (Fig. 2B). Intra-op EGD demonstrated healthy mucosa without evidence of devascularization or full-thickness perforation. Multiple biopsies/resection specimens were obtained, surgical drains placed, and a 22-French Stamm gastrojejunostomy tube for gastric decompression and distal feeding access was placed. The patient’s postoperative course was notable for delayed return of bowel function and a leukocytosis which prompted a CT abdomen pelvis with PO contrast obtained on POD 6, which was without evidence of a leak but did demonstrate an evolving fluid collection/hematoma at her gastric wall and a small subfascial abscess requiring midline staple removal and PO antibiotics. She was ultimately discharged on POD 12.

The final surgical pathology report from the peri-colonic biopsy and gastric tissue excision demonstrated fibroadipose tissue with marked reactive changes while a larger soft tissue mass with intertwined hemorrhage as well as resected omentum were both found to be involved by synovial sarcoma. Six weeks postoperatively, she resumed chemotherapy with trabectedin 1.5 mg/m^2^ Q3 weeks. The complete timeline of her treatment course is summarized in Fig. 2C.

## Discussion and conclusions

Here, we describe a case of upper extremity synovial sarcoma with metastasis to a rarely described location: the stomach. In the above case, it remains unclear if the mass abutting the stomach was exophytic and gastric in origin or centered in the mesentery, in close proximity to and eroding into the stomach. Regardless, there are very few studies describing metastatic synovial sarcoma and no studies describing metastasis to the stomach causing hemoperitoneum and hemodynamic instability, highlighting the uniqueness of this patient’s presentation.

To help counsel patients as to the natural history of synovial sarcoma, identifying prognostic features of the primary tumor and associated risk of metastasis may help frame decisions about treatment courses both at time of diagnosis and during periods of surveillance. Unfortunately, there is little consensus as to important prognostic factors in synovial sarcoma. From a histologic standpoint, many studies suggest that all synovial sarcomas are high grade and do not differentiate between FNCLCC grade 2 and grade 3 tumors while other studies report that tumor grade is an important prognosticator [9]. In a retrospective cohort study of 62 patients from 1968 to 1999, Krieg et al. showed that local recurrence occurred after ~3.6 years and metastases on average at ~5.7 years. 47% of patients developed metastasis with 79% located in the lungs, 11% in regional lymph nodes, and 7% in the chest wall and abdomen. There was one case of metastasis in the kidney and pancreas and one patient with metastasis to the brain and lungs. Again, tumor size (> 5 cm) was associated with worse OS but interestingly distant metastases were not significantly associated with tumor size [9]. Consistently, tumor size appears to be a prognostic factor and synovial sarcomas arising from sites other than the extremities are associated with poorer outcomes [3]. Using the surveillance, epidemiology, and results (SEER) database, Guo et al. evaluated patients with metastatic limb synovial sarcoma. In total, from 1975 to 2016, 217 patients with limb synovial sarcoma and metastasis at presentation were identified. Overall rates of metastasis were ~48%, but this study did not differentiate the location of metastasis and only noted that the majority of synovial sarcomas metastasize to the lung. With regards to prognostic factors, of the primary tumors with size data that metastasized, 61 (28%) were < 10 cm and 102 (47%) were > 10 cm suggesting that size may be an important prognostic factor. Irrespective of size, the 3-year overall survival of patients with limb synovial sarcoma that was metastatic at initial presentation was 27.2% [10], consistent with a more aggressive tumor biology and poorer prognosis.

Intrinsic tumor biology may also play a role in prognostication; however, the prognostic impact of SS18-SSX1 or SS18-SSX2 is unclear with two large studies showing conflicting results [11, 12]. In the case presented above, FNA of the peri-gastric hematoma/tumor demonstrated small round blue cells in abundant myxoid matrix which did not appear cytologically similar to the patient’s primary synovial sarcoma. This may be due to a sampling bias with the FNA or significant de-differentiation of the metastatic tumor which appears to have lost cytokeratin staining. Histologically, the final surgical resection specimens were strongly felt to be consistent with synovial sarcoma. One limitation of this interpretation is that the peri-gastric resection specimen was never sent for molecular diagnostics to confirm the presence of the SS18/SSX1 fusion which would have more definitively characterized this mass as a metastasis arising from the upper extremity primary. Future studies using next-generation sequencing and single-cell resolution techniques will assuredly help further characterize phenotypic and genotypic differences between primary and metastatic synovial sarcomas to help elucidate pathways and mutations more commonly associated with establishment of metastatic tumors and potentially provide new avenues for targeted therapeutic agents.

Until then, clinicians must continue to closely follow patients with soft tissue sarcomas for evidence of metastatic disease. Unfortunately, guidelines for routine locoregional surveillance as well as recommendations for surveillance of distant metastatic disease are lacking. A survey study from 2016 by Greenberg and Crawford queried practice patterns in 38 of 193 members of the Musculoskeletal Tumor Society (MSTS) and identified surgical margin, histologic grade, and tumor size as factors perceived to increase risk of local recurrence the most. However, there was no comment on best practices for surveillance of distal metastasis [13]. The NCCN provides some guidelines for routine surveillance including a history and physical every 3–6 months for 2–3 years following initial intervention (i.e., surgical resection), then every 6 months for the next 2 years and then annually. Chest imaging and periodic imaging of primary site based on estimated risk of locoregional recurrence is also recommended.

Given the propensity of the majority of soft tissue sarcomas to metastasize to the lung [9], surveillance imaging tends to focus on pulmonary imaging with chest-ray or CT scans the mainstays of routine imaging. Interestingly, in a prospective randomized single-center noninferiority trial comparing chest x-ray to chest CT, Puri et al. demonstrated that 3-year overall survival in the chest X-ray cohort was 67 vs. 66% in the chest CT cohort suggesting that detection of recurrence by chest X-ray may be adequate from a disease monitoring standpoint. This study also found that 90% of local recurrences were identified by patient self-exam [14]. Unfortunately, there is no chest X-ray equivalent for monitoring disease progression in the abdomen and cross-sectional imaging would be required for routine surveillance. In the case of the previously described patient, she had no symptoms prior to the day of presentation to suggest intra-abdominal pathology or warrant a CT of the abdomen and pelvis which raises the question of whether or not patients with high-risk features may benefit from surveillance imaging of the abdomen and pelvis. To that effect, the role of PET/CT in evaluating soft tissue sarcomas continues to evolve. The American College of Radiology appropriateness criteria for follow-up of malignant or aggressive musculoskeletal tumors from 2015 states that, “FDG-PET/CT continues to be an area of robust growth and research, with current evidence supporting its use as a problem-solving tool in equivocal cases of local or distant recurrence detected on MRI or CT.” It does not specifically comment on the use of FDG-PET/CT imaging for evaluation of metastatic disease to intra-abdominal locations.

In conclusion, we describe a unique case of synovial sarcoma of the upper extremity with peri-gastric metastasis resulting in hemoperitoneum without evidence of gastric mucosal violation. In summarizing this case, we also highlighted neo-adjuvant chemoradiation approaches for upper extremity soft tissue sarcomas such as synovial sarcoma and outlined operative approaches for management of active extravasation resulting from metastatic tumors in the lesser sac. Important primary tumor prognostic factors were reviewed and areas for future basic science studies focusing on the molecular pathogenesis of primary and metastatic soft tissue sarcomas as well as clinical studies aimed at better identifying patient populations which may benefit from expanded screening approaches were also discussed.

## Data Availability

Not applicable.

## Abbreviations

EGD: Esophagogastroduodenoscopy
EUS: Endoscopic ultrasound
FNA: Fine-needle aspiration
FNCLCC: Fédération nationale des centres de lutte contre le cancer
GIST: Gastrointestinal stromal tumor
NCCN: National Comprehensive Cancer Network
FDG-PET: Fluorodeoxyglucose-positron emission tomography
CT: Computerized tomography
